# The incidence of thyroid gland invasion in advanced laryngeal squamous cell carcinoma

**DOI:** 10.1016/j.bjorl.2019.11.003

**Published:** 2019-12-09

**Authors:** Hadi A. Al-Hakami, Mohammed A. Al Garni, Haya AlSubayea, Yazeed AlOtaibi, A. Neazy, W. Jameel, Mohammed Albouq, A. Alnufaie, Nawaf Fatani

**Affiliations:** King Saud Bin Abdulaziz University for Health Sciences, King Abdullah International Medical Research Center, Department of Surgery, Section of Otolaryngology-Head & Neck Surgery, Jeddah, Saudi Arabia

**Keywords:** Larynx, Laryngectomy, Squamous cell carcinoma, Thyroid gland, Thyroidectomy

## Abstract

**Introduction:**

Invasion of the thyroid gland is not a general feature of advanced laryngeal carcinoma. There is no need for performing thyroidectomy in all total laryngectomy cases.

**Objectives:**

To evaluate the frequency of the thyroid gland invasion in patients with advanced laryngeal squamous cell carcinoma submitted to total laryngectomy and thyroidectomy and to determine whether clinical and pathological characteristics of laryngeal carcinoma can predict glandular involvement.

**Methods:**

A retrospective case series with chart review, from March 2009 to January 2018, was undertaken in the the Princess Norah Oncology Center, King Abdul-Aziz Medical City, Jeddah/KSA. An inception cohort of 56 patients with laryngeal squamous cell carcinoma was considered. Nine cases were excluded. All patients had advanced stage cancer of the larynx (clinically T3‒T4) and underwent total laryngectomy in association with thyroidectomy. Total thyroidectomy was performed in all bilateral lesions or if there was suspicion of contralateral lobe involvement. Hemithyroidectomy was performed in all lateralized lesions. Retrospective histopathologic analysis of thyroid specimens was subsequently performed. The frequency of thyroid gland invasion was calculated and analysis of demographic, clinical and pathological characteristics associated with thyroid gland invasion was performed.

**Results:**

In all, 47 patients underwent total laryngectomy (40 treated with primary laryngectomy and seven treated with salvage laryngectomy following radiation failure or chemoradiation failure). Hemithyroidectomy was performed in 42 patients and the total thyroidectomy was performed in five patients. The overall frequency of invasion of the thyroid gland was 4.3%. Glandular involvement was seen in one advanced transglottic squamous cell carcinoma and one subglottic. In spite of thyroid cartilage invasion in 25.5% of cases detected in the preoperative radiological imaging, only one case demonstrated microscopic thyroid gland invasion.

**Conclusions:**

Thyroidectomy may only be required during total laryngectomy for selected cases of advanced transglottic tumors and tumors with subglottic extension more than 10 mm.

## Introduction

Total laryngectomy is the standard of care for operable advanced (T3 and T4) squamous cell carcinoma of the larynx. Advanced squamous cell carcinoma (SCC) of the larynx has the potential to invade the thyroid gland. This invasion occurs mainly by direct extension due to the proximity of the thyroid gland to the larynx or by lymphovascular spread.^1^, ^2^, ^3^, ^4^ Invasion of the thyroid gland is a stage T4a under AJCC TNM classification (8th Edition), the group staged IVa (moderately advanced local disease), unless distant metastasis has occurred. The 3 and 5 years Disease-Free Survival (DFS) for stage IV cancers is approximately 40–45% and 30–35%, respectively.^5^ The frequency of cancer involvement of the thyroid gland in advanced SCC of the larynx varies in the literature between 1% and 30%.^6^, ^7^, ^8^ According to the risk variability, the decision to surgically address the ipsilateral vs. total thyroidectomy as part of the surgical treatment of all such cases remains controversial.^2^, ^3^, ^7^, ^8^ A recent meta-analysis study by Mendelson et al. has advised ipsilateral hemithyroidectomy for all tumors with subglottic extension greater than 10 mm, as well as all transglottic tumors.^9^ Adding thyroidectomy to the surgical treatment of laryngeal cancer increases the risks of hypothyroidism and hypoparathyroidism to 23–63% and 25–52%, respectively.^10^, ^11^, ^12^, ^13^ The incidence increases to 70–91% when adjuvant radiotherapy is given post total laryngectomy.^14^ Hypothyroidism and hypoparathyroidism may need lifelong physician care and the cost of care for these patients increases significantly.^15^ Currently, there is no consensus on the guidelines directing the management of the ipsilateral thyroid lobe during total laryngectomy for laryngeal squamous cell carcinoma. A definition of the clinical and pathological features associated with thyroid involvement would be of great value in such cases. This definition could direct surgical treatment and reduce morbidities and improve quality of life without impairing treatment objectives.^9^, ^16^ This study aimed to evaluate the frequency of thyroid gland invasion in patients with advanced SCC of the larynx undergoing total laryngectomy associated with hemithyroidectomy or total thyroidectomy and to determine whether clinical and pathological features can predict thyroid gland invasion.

## Methods

### Ethical approval

Approval to conduct a retrospective review of the medical records of patients was obtained from the institutional review board of the institutional review board of King Abdullah International Medical Research Center (Reference nº IRBC/2168/18).

### Study and settings

Medical records of 56 patients who underwent total laryngectomy for advanced stage laryngeal squamous cell carcinoma from March 2009 to March 2018 were retrospectively reviewed. Patients with incomplete record data, along with patients undergoing surgery outside the the Princess Norah Oncology Center, King Abdul-Aziz Medical City, rare laryngeal tumors such as chondrosarcoma, distant metastasis and patients who did not undergo thyroidectomy were excluded. A total of nine cases were excluded from the study. The retrieved records were inspected for details of demographic profile, clinical examination, radiological imaging, operative notes, and histopathological details. We staged the cases according to the American Joint Committee on Cancer, 8th edition. Tumor extent, subsite involved, cervical lymphadenopathy, and thyroid gland involvement were evaluated based on the histological analysis of pathological specimens retrospectively. Subglottic invasion was considered when there was an extension of the tumor or primary involvement by the tumor of the region located more than 10 mm below the true vocal folds. Pre and postoperative therapies received by the patients were also noted. Management protocol involved surgical excision of the thyroid gland in association with total laryngectomy, indicated for patients with advanced SCC of the larynx when there is evidence of tumor extension into the subglottic region and extra-laryngeal tissues. Unilateral thyroidectomy was performed in all lateralized cancers. All patients with bilateral palpable or suspicious thyroid lesions on examination and radiological imaging underwent total thyroidectomy. The data was entered and analyzed using the Statistical Package for the Social Sciences SPSS software version 21.0 (SPSS Inc., Chicago, IL, USA).

## Results

Of the 47 patients included 39 (83%) patients were male and 8 (17%) were female. Their age ranged from 30 to 85 years. The mean patient age (±SD) was 63.6 ± 13.5 years. 36 (76.6%) patients had a current or past history of smoking. The cancer was squamous cell carcinoma in all patients. According to clinical and radiological imaging assessment the tumor was located in the supraglottic region in 4 (8.5%), glottic in 16 (34%), subglottic in 2 (4.3%), and transglottic in 25 (53.2%) cases. On radiological imaging (Computerized Tomography and Magnetic Resonance Imaging) thyroid cartilage invasion was evident in 12 cases (25.5%), suspicious thyroid gland invasion in 4 (8.5%) cases, and 11 patients (23.4%) had positive lymph nodes involvement as shown in Table 1. The postoperative Tumor-Node-Metastasis (TNM) staging was reported, 37 (78.7%) of the cases were stage IVa and 10 (21.3%) were stage III (Table 2). The details of postoperative TNM staging and pathological data are shown in Table 2. All patients underwent total laryngectomy, either primary in 40 (85.1%) cases or salvage in 7 (14.9%) cases with hemithyroidectomy or total thyroidectomy after thorough discussion in the tumor board. According to the management protocol hemithyroidectomy was performed in 41 (87.2%) of the cases, and total thyroidectomy in the remaining 6 (12.8%) cases. Cervical lymph node dissection either modified or radical was a part of all performed surgeries. More details about surgical settings are shown in Table 3. Postoperatively all patients were subsequently reviewed by the tumor board team to address the need for adjuvant treatment. However, 34/40 (85%) of primary laryngectomy cases went on to receive external beam radiation therapy alone and 3 (7.5%) received chemoradiotherapy. In the remainder of the patients, 2 (5%) were not medically fit to receive adjuvant therapy, and 1 (2.1%) did not require adjuvant therapy. On review of the pathological specimens, thyroid gland invasion by squamous cell carcinoma was identified in 4.3% (2/47) patients. 16.7% (1/6) patients in the total thyroidectomy group had squamous cell carcinoma involving the thyroid lobe, extending from a subglottic lesion. In the hemithyroidectomy group, 2.4% (1/41) had squamous cell carcinoma involving the thyroid lobe, extending from a transglottic lesion with thyroid cartilage involvement (Table 4). No patients with glottic including anterior commissure or supraglottic obstructing lesions had direct extension into the thyroid gland. 1 out of 2 patients with thyroid gland invasion had suspicious thyroid gland involvement in the radiological imaging. Interestingly, two of hemithyroidectomy group had incidental papillary carcinoma.

**Table 1:** Preoperative clinical and radiological imaging data.

Variable	n (%)
Glottis	16 (34%)
Supraglottic	4 (8.5%)
Subglottic	2 (4.3%)
Transglottic	25 (53.2%)
Anterior commissure involvement	5 (10.6%)
Thyroid cartilage involvement	12 (25.5%)
Suspicious thyroid gland involvement	4 (8.5%)
Lymph node involvement	11 (23.4%)

Glottic in 16 (34%) and transglottic in 25 (53.2%) cases were major one in this study.

**Table 2:** Postoperative pathological data.

Variable	n (%)
Primary Tumor Staging	
T3	10 (21.3)
T4a	37 (78.7%)
N Staging	
N0	34 (72.3%)
N1	5 (10.6%)
N2a	4 (8.5%)
N2b	2 (4.3%)
N2c	2 (4.3%)
N3	0 (0%)
M staging	
M0	47 (100%)
M1	0 (0%)
Global TNM Staging	
III	10 (21.3)
IVa	37 (78.7%)
IVb	0 (0%)
Surgical Margins	
Positive	1 (2.1%)
Negative	46 (97.9%)
Extracapsular Extension	
Positive	3 (6.4%)
Negative	44 (93.6%)
Thyroid Gland Invasion	
Positive	2 (4.3%)
Negative	45 (95.7%)

TNM classification according to American Joint Committee on Cancer (AJCC) criteria 8th edition.

**Table 3:** Comparison of surgical settings for all patients.

Variable	n
Primary laryngectomy (n = 40)	
EBRT (postoperative)	35
EBRT + chemotherapy (postoperative)	3
No additional intervention	2
Salvage laryngectomy (n = 7)	
Radiation failure	4
Radiation and chemotherapy failure	3
Total thyroidectomy (n = 6)	
Primary laryngectomy	2
Salvage laryngectomy	4
Hemithyroidectomy (n = 41)	
Primary laryngectomy	38
Salvage laryngectomy	3

EBRT, External Beam Radiation Therapy.

Primary Laryngectomy (Hemithyroidectomy was the major procedure performed in our settings.

**Table 4:** Pathologic analysis of thyroid gland specimens found to have squamous cell carcinoma correlated with primary sites of tumor.

Variable	n
Total Thyroidectomy (n = 1)	
Subglottic	1
Hemihyroidectomy (n = 1)	
Transglottic with thyroid cartilage involvement	1

## Discussion

Ogura first described thyroid gland involvement as a metastatic feature of laryngeal cancer in 1955, recommending its routine removal to ensure adequate local control of the tumor.^17^ Advanced laryngeal squamous cell carcinoma has the potential to invade the thyroid gland through direct extension due to specific anatomical considerations or lymphatic and hematogenous spread. Brennan et al. found the majority of thyroid gland invasion by laryngeal squamous cell carcinoma (78%) was through direct extension, with only 10% (11/107) of cases by the lymphatic spread.^18^ In such cases, thyroid gland invasion is a poor prognostic factor.^11^, ^16^, ^17^ Biel et al. have reported recurrence in all their cases of thyroid gland invasion within ten months of definitive treatment.^10^ Laryngeal regions most susceptible to spread are at the anterior angle of the thyroid cartilage, the anterior commissure and the cricothyroid membrane.^19^, ^20^ Typically, perichondrium provides an excellent protective barrier to the spread of carcinoma. However, once the carcinoma has spread beyond the perichondrium, it may spread in a subperichondrial plane throughout the cartilage, further predisposing to extralaryngeal spread.^21^ A defect exists in the inner perichondrium, at the anterior angle of the thyroid cartilage, where it is pierced by Broyles’s ligament, providing a route for tumor spread.^20^, ^22^ The frequencies of thyroid gland invasion by advance laryngeal SCC found in the literature range from 1% to 30%.^3^, ^6^, ^7^, ^11^^,^^23^ The frequencies found in the presented study suggest that thyroid surgery would be performed unnecessarily in the majority of cases. It is known that the performance of thyroidectomy, whether partial or total, greatly increases the morbidity as hypothyroidism and hypoparathyroidism. Lo Galbo et al., for example, showed that of 37 patients who underwent total laryngectomy with hemithyroidectomy in their series, 78.3% developed hypothyroidism within five year follow up period.^24^ The indications for thyroidectomy as part of the treatment of advanced laryngeal carcinoma have been thoroughly discussed in the literature. Within our study, only 2/47 (4.3%) cases of thyroid gland invasion were detected based on the permanent pathology; one case with advanced primary subglottic SCC and the other case with advanced transglottic SCC with thyroid cartilage invasion. There was no association of thyroid gland invasion with lymph node staging or with microscopic analysis of tumors as the presence of angiolymphatic or perineural invasions. These findings support the theory that glandular invasion by advance laryngeal SCC occurs most commonly by contiguity or direct invasion. Similar results were observed by João Mangussi-Gomes et al. study.^25^ A meta-analysis conducted by Mendelson et al. showed that only transglottic or subglottic tumors or tumors with subglottic extension >10 mm were associated with invasion of the thyroid gland.^9^ In the series by Biel and Maisel, they found that 70% of all glottic cancers with thyroid gland involvement had subglottic extension more than 1.5 cm.^10^ Sparano et al. determined that 100% (7/7) of cases with thyroid gland involvement had subglottic extension beyond 15 mm.^2^ In a series by Yuen et al., 16% of tumors with subglottic extension demonstrated thyroid gland involvement.^26^ Predisposition of subglottic tumors to thyroid gland involvement may be due to direct extension through the paraglottic space or through the gaps between tracheal rings or by lymphatic spread.^19^, ^27^ Transglottic lesions, as well as paraglottic space involvement, have been further predictive of extralaryngeal spread. In a series by Mourad et al., 71% (5/7) of cases with thyroid gland invasion were transglottic lesions.^28^ Harrison showed an incidence of 25% involvement of thyroid gland in a series evaluating the tumor spread of transglottic and subglottic tumors.^29^ Thyroid cartilage involvement has also been considered a risk factor for likely thyroid gland involvement and an indication for thyroid lobectomy.^2^, ^9^ In our series in spite of 12/47 (25.5%) patients had thyroid cartilage invasion in the pre-operative radiological imaging, only 8.3% (1/12) patients had microscopic thyroid gland invasion which was a case of advance transglottic carcinoma.

This study has inherent limitations in that it is a retrospective study, based on the analysis of medical and pathological study reports. In a retrospective study, generally selection bias occurs if selection of exposed and non-exposed subjects is somehow related to the outcome. A retrospective study has its own limitations and is subject to numerous threats to validity, which limit the interpretation and generalizability of the results. However these are true when the studies are interventional studies in process to establish a cause-and-effect relationship with one or more outcomes. Most of the problems with misclassification occur with respect to exposure status, not outcome. In our study most of the information was from chart review, and established diagnosis reconfirmed by retrospective histopathologic analysis. Analysis of demographic, clinical and pathological characteristics associated with thyroid gland invasion was performed. Hence in absence of any intervention we did not face any major limitations which have effect on the outcome of studies and statistical analysis. Moreover, the small number of patients with thyroid gland involvement in our sample affected proper methodology of analyzing the degree of association between involvement of the thyroid gland and other variables by calculating the Odds Ratio (OR) and its 95% Confidence Interval (95% CI). Studies with larger samples are needed to better define indications for thyroidectomy in the context of laryngeal squamous cell carcinoma.

## Conclusion

Invasion of the thyroid gland is not a general feature of advanced laryngeal squamous cell carcinoma. There is no need for performing thyroidectomy in all total laryngectomy cases. The thyroidectomy may only be required during total laryngectomy for selected cases of advanced transglottic tumors and tumors with subglottic extension more than 10 mm.

## Conflicts of interest

The authors declare no conflicts of interest.
